# Supplementary figures and images for: Cell-type-specific control of secondary cell wall formation by Musashi-type translational regulators in Arabidopsis
Source: eLife. 2023 Sep 29;12:RP88207. doi: 10.7554/eLife.88207 (PMC10541177; doi:10.7554/eLife.88207)

Figure 1- figure supplement 2B:

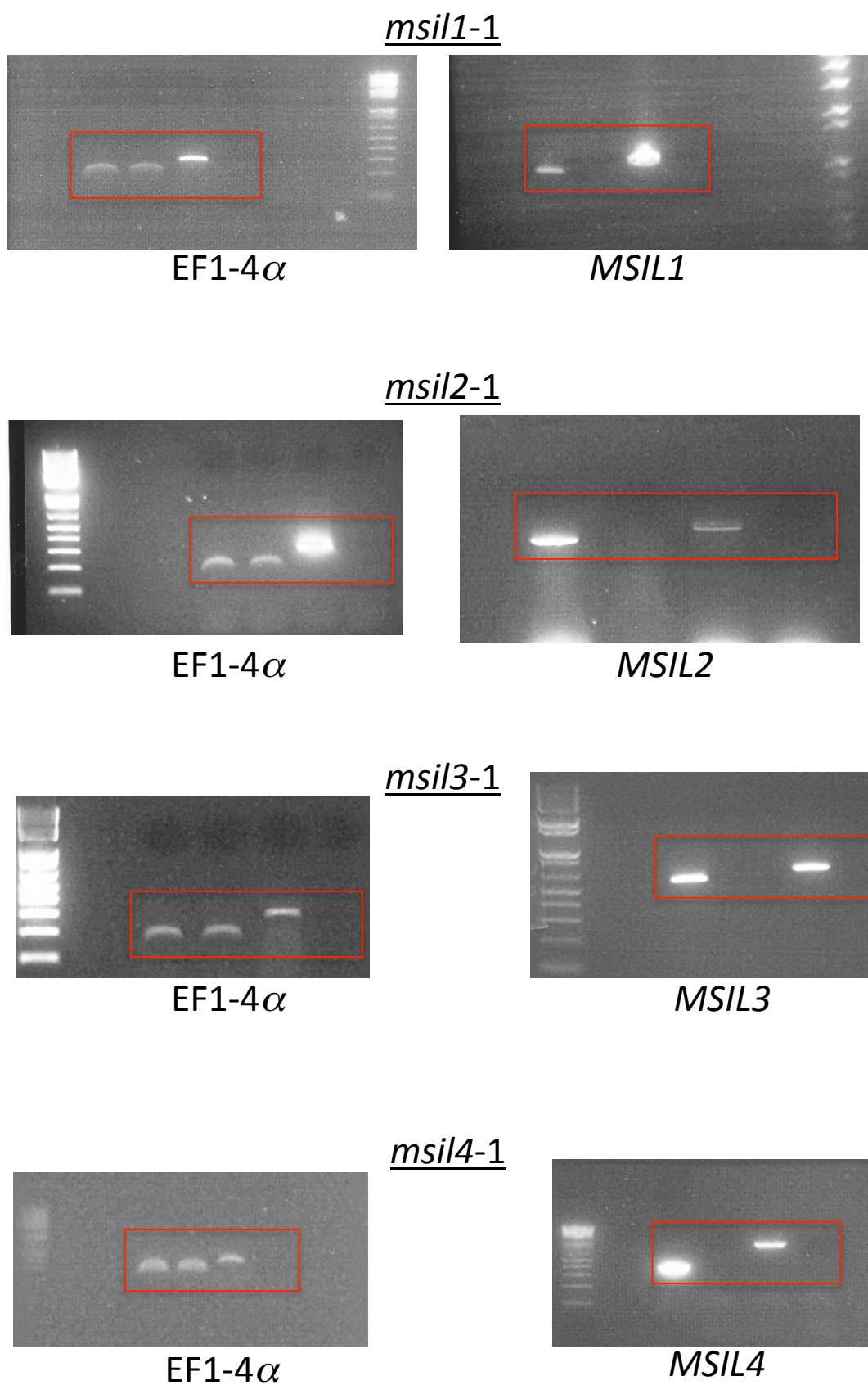

Supplement: Figure 1—figure supplement 2—source data 1. — MSIL1,2,3,4 specific primers were used to characterize and validate the nature of the insertion lines. Primers corresponding to EF1-4α gene were used to confirm the equilibration. [file elife-88207-fig1-figsupp2-data1.zip › data_RT_Figure 1 supplement 2.pdf]

Figure 1- figure supplement 2C:

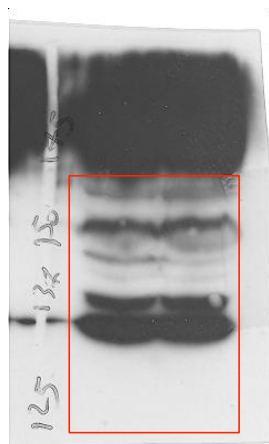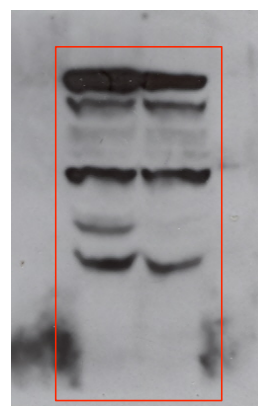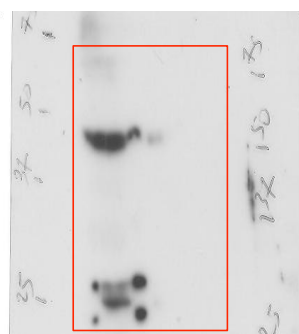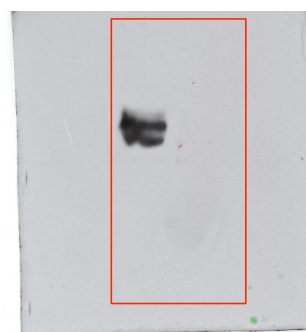

Supplement: Figure 1—figure supplement 2—source data 2. — Anti MSIL1,2,3,4 antibodies were used for the immunoblotting analysis. [file elife-88207-fig1-figsupp2-data2.zip › Data_WB_Figure 1 supplement figure 2C.pdf]

Figure 1- figure supplement 3D:

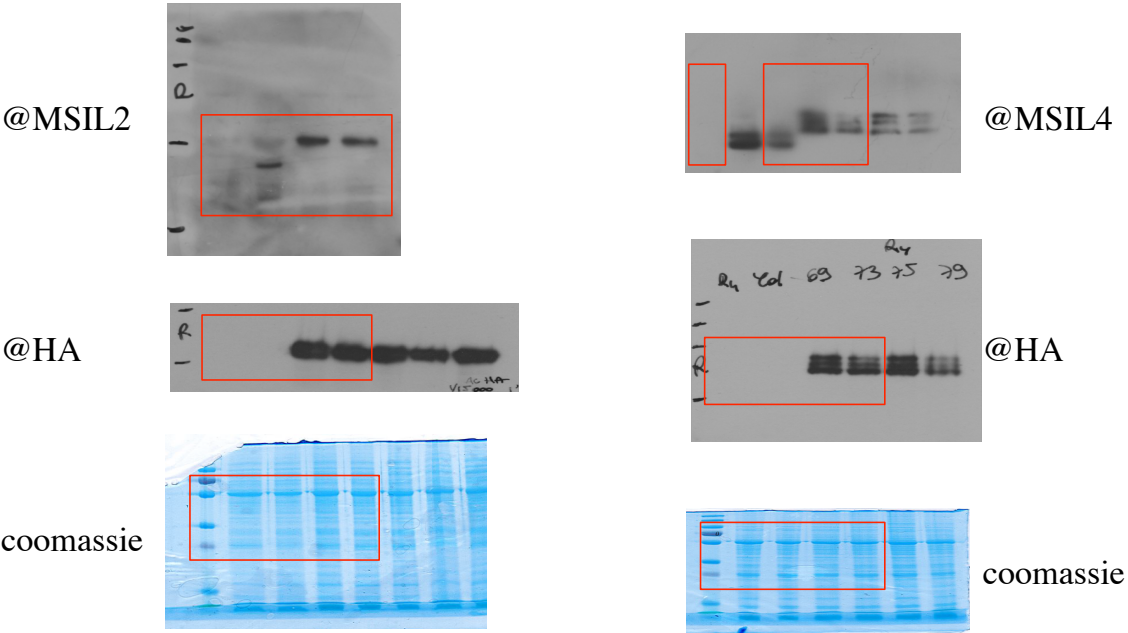

Supplement: Figure 1—figure supplement 3—source data 1. — Western blots were performed using either endogenous MSIL4 (top) or anti-HA (bottom) antibodies. Coomassie gels are used as protein loading controls. [file elife-88207-fig1-figsupp3-data1.zip › Data_WB_Figure 1 supplement Figure 3D.pdf]

Figure 2C

MSIL4

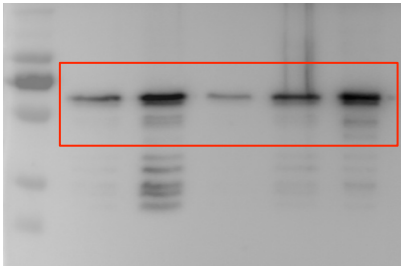

MSIL4<sup>RRM</sup>

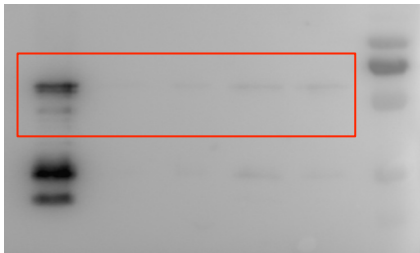

Supplement: Figure 2—source data 1. — Western blots were performed using anti-His antibodies. [file elife-88207-fig2-data1.zip › Data_WB_Figure 2.pdf]

Figure 6D

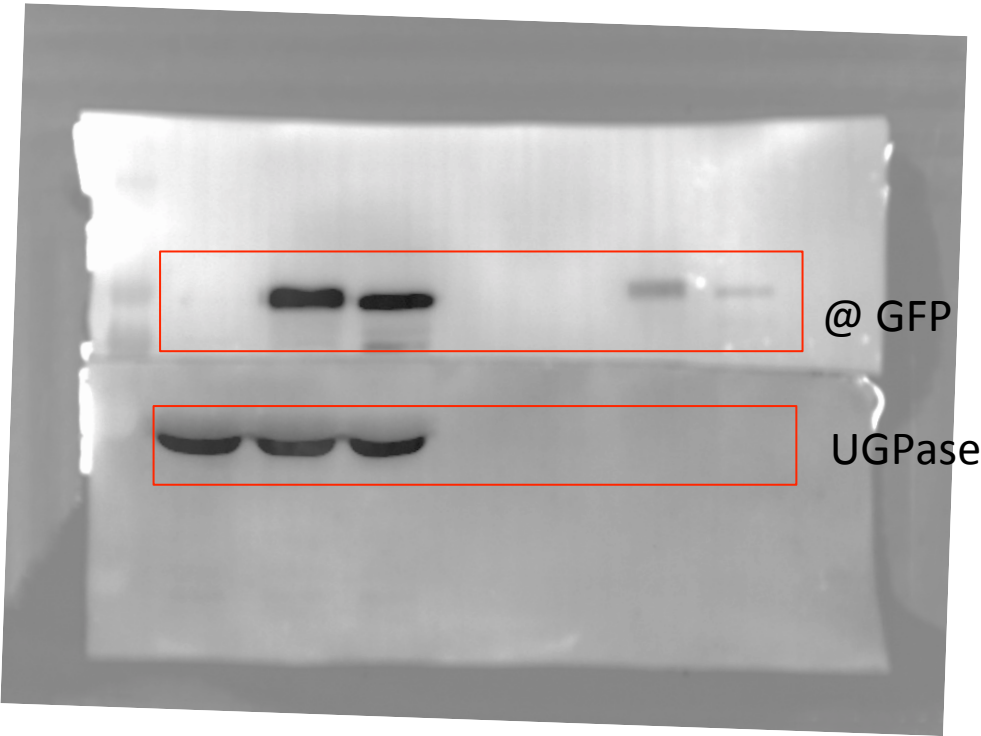

Supplement: Figure 6—source data 1. — Western blots were performed using either the antiGFP or antiUGPase antibodies. [file elife-88207-fig6-data1.zip › Data_WB_Figure 6.pdf]

Figure 6D

Cut in Figure 6D to fit with the western image

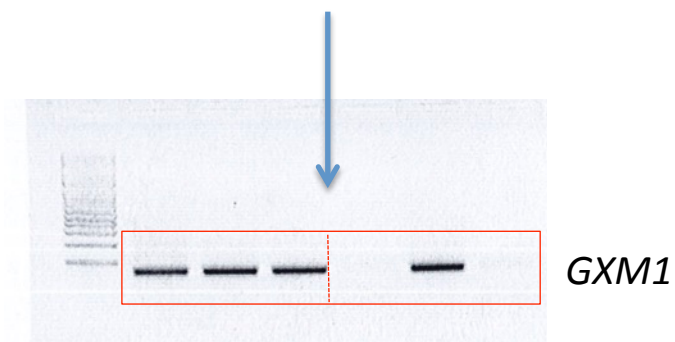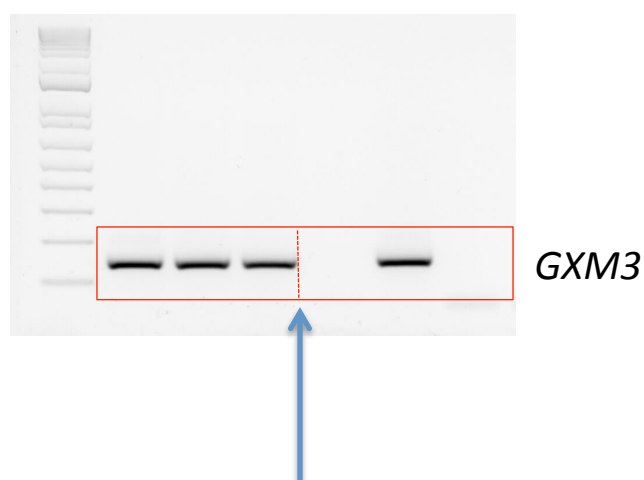

Cut in Figure 6D to fit with the western image

Supplement: Figure 6—source data 2. — GXM1 and GXM3-specific primers were used on the various RNA fractions. [file elife-88207-fig6-data2.zip › Data_RT_Figure 6.pdf]
